# Involvement of Nrf2 in Ocular Diseases

**DOI:** 10.1155/2017/1703810

**Published:** 2017-03-27

**Authors:** Shehzad Batliwala, Christy Xavier, Yang Liu, Hongli Wu, Iok-Hou Pang

**Affiliations:** ^1^Texas College of Osteopathic Medicine, University of North Texas Health Science Center, Fort Worth, TX 76107, USA; ^2^Department of Pharmaceutical Sciences, University of North Texas Health Science Center, Fort Worth, TX 76107, USA; ^3^North Texas Eye Research Institute, University of North Texas Health Science Center, Fort Worth, TX 76107, USA

## Abstract

The human body harbors within it an intricate and delicate balance between oxidants and antioxidants. Any disruption in this checks-and-balances system can lead to harmful consequences in various organs and tissues, such as the eye. This review focuses on the effects of oxidative stress and the role of a particular antioxidant system—the Keap1-Nrf2-ARE pathway—on ocular diseases, specifically age-related macular degeneration, cataracts, diabetic retinopathy, and glaucoma. Together, they are the major causes of blindness in the world.

## 1. Introduction

Free radicals such as reactive oxygen species (ROS) are oxidants that are normally produced as a by-product of normal aerobic metabolism [1]. In addition to endogenous and metabolic production of ROS, environmental sources such as light, smoke, and heavy metals also contribute to increased ROS burden [2–7]. An excessive level of ROS damages many cellular and tissue components. In the eye, these can adversely affect vision. Antioxidants are molecules that reduce ROS and keep the oxidative damage at a minimum. In the human cell, antioxidant molecules include both nonenzymatic compounds such as glutathione (GSH), thioredoxin (Trx), ascorbate, *α*-tocopherol, *β*-carotene, and coenzyme Q10 and enzymes such as catalase, glutathione peroxidase, and superoxide dismutase (SOD) [8]. Antioxidant systems in the cell can be categorized into two consecutive phase reactions: phase I and phase II reactions [9–11]. Phase I reactions are reduction-oxidation (redox) reactions carried out via the cytochrome P450 enzyme system. Phase I products are then conjugated with hydrophilic molecules such as GSH in the phase II reactions. The molecules that catalyze phase II reactions can be further classified as “direct” or “indirect.” “Direct” antioxidants include SOD1 and GSH, which directly quench ROS. “Indirect” antioxidants serve to synthesize and recycle GSH and Trx, which are the two most important ROS quenchers. Imbalance between oxidants and antioxidants in favor of oxidants leads to oxidative stress. This imbalance has at least two direct consequences: damage to individual components of the living cell and induction of inflammatory pathways that further perpetuate the damage. Oxidative stress can denature lipids and proteins [10, 12–14], as well as induce DNA and RNA fragmentation [15–17], leading to cell dysfunction, injury, and death. In addition, an excess of oxidative free radicals also increases the expression of proinflammatory genes and activates the inflammation process [18, 19]. Inflammation often exacerbates the oxidative stress, creating a self-perpetuating, vicious cycle of oxidation and inflammation [20, 21].

**Figure 1:**
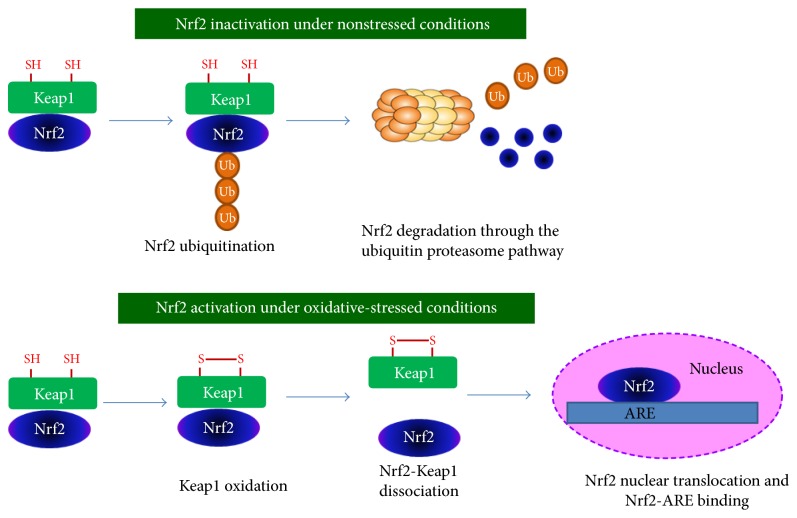
Proposed molecular mechanisms of oxidative stress-induced Nrf2 activation. Under nonstressed conditions, Keap1 keeps Nrf2 sequestered in the cytosol, where it mediates proteasomal degradation of Nrf2. Under oxidative-stressed conditions, cysteine residues of Keap1 are oxidized, forming a disulfide bridge. Oxidized Keap1 dissociates from Nrf2, allowing Nrf2 to translocate to the nucleus, bind to the ARE region, and initiate transcription of target genes.

The Keap1-Nrf2-ARE pathway plays a critical role in the regulation of a comprehensive and protective antioxidant response [22]. Nuclear factor erythroid-2-related factor 2 (Nrf2) is a transcription factor that is upregulated in times of oxidative stress. It puts in place a sequence of events that ultimately protect the cell from oxidative injury. Nrf2 activates transcription of antioxidant enzymes by binding to the antioxidant response element (ARE) in the promoter regions of its target genes [23, 24]. As shown in Figure 1, in the absence of oxidative stress, Kelch-like ECH-associated protein 1 (Keap1) keeps Nrf2 sequestered in the cytosol, where it mediates proteasomal degradation of Nrf2 [25–27]. Upon exposure to ROS, Keap1 undergoes a conformational change that allows Nrf2 to translocate to the nucleus, bind to the ARE region, and initiate transcription of target genes [24].

Both “direct” and “indirect” antioxidant enzymes are regulated by Nrf2. “Direct” enzymes that are directly regulated by Nrf2 include catalase and SOD1, and the “indirect” enzymes include GSH- and Trx-generating enzymes and heme oxygenase- (HO-) 1 [28–30]. It is important to note that the baseline expression of many antioxidant enzymes is not significantly upregulated by Nrf2. Rather, the main function of Nrf2 is to induce the antioxidant response [28–30]. This antioxidant response induced by Nrf2 has an early acute phase mediated by the “direct” enzymes and a late chronic phase mediated through maintenance of GSH and Trx. Since the depletion of GSH is directly correlated to oxidative injury and cell death via apoptosis, Nrf2 thus serves as an important link between cell survival and antioxidant gene expression [28–30].

## 2. Oxidative Stress and Nrf2 in Ocular Diseases

The eye is a prominent target of oxidative stress. It is continuously exposed to various oxidative conditions, such as photo-oxidation, ionizing radiation, smoke, and various forms of pollutants. The retina in particular, because of its high metabolic activities, is a highly perfused and oxygenated tissue. It also contains higher concentrations of polyunsaturated fatty acids than other tissues in the human body [31]. The combination of these factors renders it vulnerable to injurious actions of oxidants such as ROS. Thus, oxidative stress has been associated with many ocular disorders, notably age-related macular degeneration (AMD), cataract, diabetic retinopathy (DR), and glaucoma [32–34]. This review summarizes the current understanding of contributions of oxidation and the Keap1-Nrf2-ARE signaling system in these diseases.

### 2.1. Age-Related Macular Degeneration

#### 2.1.1. Age-Related Macular Degeneration: Leading Cause of Irreversible Blindness

Age-related macular degeneration is the leading cause of irreversible blindness in individuals aged 60 years and over in developed countries [35]. Due to the increase in the aging population, AMD has become a major healthcare concern, affecting nearly 25 million people worldwide [35]. AMD is often linked to deterioration of central vision, blurriness, and in advanced cases, permanent vision loss. Drusen are extracellular deposits of debris that accumulate between RPE and Bruch's membrane. The appearance of drusen is considered as the clinical hallmark of AMD. Although the drusen deposits may not initially cause vision loss, individuals with drusen are associated with a greater risk of developing the advanced forms of AMD [36]. Crabb et al. developed a method for isolating drusen for proteome analysis. The authors identified many forms of protein oxidative modifications, including cross-linked species of tissue metalloproteinase inhibitor 3 and vitronectin, and carboxyethyl pyrrole protein adducts in drusen [37, 38]. These observations suggest that protein oxidative modifications may contribute to drusen formation. Another hallmark of AMD is the accumulation of lipofuscin in RPE cells. Lipofuscin, also called “age pigment,” is often considered as a symbol of aging [39, 40]. Lipofuscin granules in the RPE are autofluorescent aggregates which are caused by the lifelong accumulation of the nondegradable end products from the phagocytosis of photoreceptor outer segments. Age-dependent accumulation of lipofuscin may cause RPE damage and thereby is associated with AMD pathogenesis.

AMD has two distinct forms: dry (nonexudative) and wet (exudative) forms. The wet form, which is characterized by choroidal neovascularization (CNV)—an abnormal, vascular endothelial growth factor- (VEGF-) dependent development of defective blood vessels below the retina, affects approximately 10% of AMD cases in the US. The use of anti-VEGF antibodies or VEGF-binding peptides is a recently developed therapy; this approach reduces the proliferation of new blood vessels and alleviates the symptoms of the disease [41]. The dry form, characterized by geographic atrophy, is more prevalent, affecting 90% of AMD patients [42]. Its pathogenesis is relatively unknown, and currently, there is no known cure. Interestingly, the Age-Related Eye Disease Study has shown that an antioxidant and mineral cocktail containing *β*-carotene, vitamin C, vitamin E, and zinc is effective in slowing down the progression of dry AMD by 25%–30% over a 5-year period [43]. This therapeutic breakthrough highlights years of research on oxidative stress as an important contributor and risk factor for AMD pathology.

#### 2.1.2. Contribution of Oxidative Stress in AMD Pathology

Because aging is a major risk factor for AMD, chronic oxidative stress is believed to be an important player in promoting the pathogenesis and progression of AMD. It has been reported that sunlight, UV exposure, cigarette smoke, complement H polymorphisms, high-fat diet, and lack of an antioxidant-rich diet can heighten the risk for individuals to develop AMD [35, 44–46]. The macula, an oval-shaped area of approximately 5.5 mm in diameter, is located at the posterior pole of the retina. In the center of the macula is the foveola (also termed fovea centralis), an area of 1.5 mm in diameter. It comprises a high density of closely packed cone photoreceptors, which provides high-acuity central vision. The retinal pigment epithelium (RPE) is a pigmented cell layer, which at the apical surface, interacts with photoreceptor outer segments of the interphotoreceptor matrix and at the basal surface, connects to the underlying choroid via the acellular Bruch's membrane [47]. RPE is involved in many functions of the retina including vitamin A metabolism, choriocapillaris maintenance, immunity, heat exchange, and forming part of the outer blood-retina barrier (BRB). Most importantly, RPE is primarily responsible for the phagocytosis of photoreceptor outer segments, thereby promoting photoreceptor health [47, 48]. Because light can be damaging and can cause oxidative stress, the photoreceptors are constantly broken down and removed by the RPE. Thus, the RPE's role in the maintenance of the macula and photoreceptors makes it a tissue heavily involved and affected by AMD pathology. Several studies have correlated AMD with chronic oxidative stress, as donor eyes have shown higher oxidative modifications of protein sulfhydryl groups, DNA, lipids, and other molecules [49]. Compared to other tissues, the retina has high metabolic rate, and as a consequence, ROS are leaked as a by-product of metabolism. The combination of a high metabolic rate and its high quantity of polyunsaturated fatty acids makes the retina more prone to ROS accumulation. Moreover, the macula receives a significant amount of blood supply, which makes it more susceptible to endogenous oxygen and oxidative stress [35, 50, 51]. However, in small amounts, ROS are vital for RPE cell signaling and function. As a homeostatic mechanism, the RPE performs continuous phagocytosis of the photoreceptor outer segments, a process that is induced by H_2_O_2_ production. Signal transduction processes are also driven by ROS signaling and may allow for downstream events that lead to the transcription and translation of proteins. Normally, the endogenous antioxidant enzymes critically reduce the total amount of ROS by directly scavenging free radicals, repairing cell damage, and restoring the redox status of the cell. For example, catalase is increased in healthy RPE cells during phagocytosis to directly scavenge H_2_O_2_ and prevent unnecessary increases in ROS. Therefore, a critical balance of ROS production and antioxidants is essential to allow for the unhindered functioning of RPE cells. As individuals age, the expression and effectiveness of their antioxidant enzymes decrease, leading to an overwhelming oxidative stress environment in the cell. When the level of ROS exceeds the capacity of the antioxidant enzymes to handle the burden, cell death can be excessive, leading to retinal degeneration. Even though the retina is equipped with numerous antioxidant enzymes, the tissue becomes highly vulnerable with age, as well as various exogenous oxidative stresses like UV light and cigarette smoke. Cigarette smoke contributes numerous pro-oxidants and is predicted to generate about 10^15^ free radicals with every puff [52]. Smoking induces inflammation and encourages the oxidation of proteins, lipids, and DNA due to the reduction of antioxidants like ascorbic acid. Furthermore, smoking has been shown to directly target RPE in a dose-dependent manner and is linked with geographic atrophy, a hallmark of the untreatable dry form of AMD [53]. In summary, with aging and chronic oxidative stress due to UV light or cigarette smoke and impaired antioxidant enzyme function, photoreceptor damage and death accelerate, drusen deposits form, and inflammation increases [54]. Because mitochondria are the primary organelles that produce ROS and free radicals as a by-product of metabolism, mitochondrial dysfunction is often linked to AMD progression [55]. This is significant because mitochondria play key roles in promoting cell survival and ATP production. Therefore, antioxidants that can quench free radicals are of particular interest due to their potential in treating oxidative stress-induced ocular diseases like AMD.

#### 2.1.3. Involvement of Nrf2 Pathway in AMD

Because of its ability to regulate multiple antioxidant enzymes, the Nrf2 pathway has been hypothesized to be involved in AMD. In multiple studies, aging has been corroborated with many oxidative stress-induced diseases, especially age-related ocular diseases [44, 54, 55]. Lenox et al. determined that the increased activation of the unfolded protein response (UPR) in the aged retinas leads to a severe decline of Nrf2 and its downstream protein HO-1. Due to the compromised antioxidant activity, proinflammatory markers such as RANTES increased, which denotes how aging plays a major role in age-related retinopathies like AMD and diabetic retinopathy [56]. A recent study by Sachdeva et al. showed that the RPE of aging mice displays higher levels of Nrf2, HO-1, and NAD(P)H:quinone oxidoreductase 1 (NQO1). However, when challenged with sodium iodate, the Nrf2 pathway fails to activate. Aged RPE also displays general signs of oxidative damage including increased malondialdehyde (MDA) and superoxide accumulation. Only by using a conditional knockout of *Keap1*, the inhibitor of Nrf2, did aged RPE have some restoration of Nrf2 signaling [57].

Mutations in Nrf2 have been associated with a higher risk of AMD development. Identified from DNA extracted from peripheral blood lymphocytes of wet and dry AMD patients, one mutation of Nrf2 at 25129A>C increases the risk for AMD. The C/C genotype showed a predilection for dry AMD, whereas having an A/C genotype decreased the likelihood of having AMD. The C/C genotype was found to be particularly detrimental when linked with age, bad dietary habits, smoking habits, and an apparent family history [58].

Autophagy, literally defined as “self-eating,” is a critical cellular self-protective mechanism that involves the lysosomal-mediated degradation of misfolded proteins, protein aggregates, and damaged organelles like the endoplasmic reticulum, mitochondria, and ribosomes. Autophagy-related proteins are found to be highly expressed and functionally active in the human retina [59, 60]. Both basic and clinical studies have revealed the connection between dysregulated autophagy and AMD. For example, Wang et al. found that drusen in AMD donor eyes contain markers for autophagy and exosomes [61, 62]. Their in vitro autophagy model also indicated that when RPE cells were stressed by mitochondrial oxidative damage, autophagy markers and exosome markers are both upregulated. They proposed that increased autophagy and the release of intracellular proteins via exosomes by aged RPE might contribute to the formation of drusen. Recently, researchers have found autophagy and the Nrf2 pathway are closely linked by the direct binding between p62 (a selective substrate for autophagy) and Keap1 [63, 64]. Both Yamamoto and Zhang's research teams reported that a deficiency in autophagy-sequestered Keap1 into aggregates through direct p62 and Keap1 binding, resulting in Nrf2-Keap1 dissociation and transcriptional activation of Nrf2 target genes. Interestingly, in aged *Nrf2* KO mice, intermediate structures of autophagy, such as the autophagosome and autolysosome, were accumulated in RPE and Bruch's membrane. This accumulation may be due to increased autophagic flux or decreased final degradation by the lysosome [65]. These data strongly suggest that Nrf2-Keap1 and autophagy act in concert to ensure protein quality control and maintain metabolic homeostasis, thereby protecting aging RPE from oxidative stress-induced degeneration.

HO-1 (*HMOX1*) and HO-2 (*HMOX2*), both downstream targets of Nrf2 that are involved in toxic heme catabolism to the antioxidant biliverdin, also have associated polymorphisms that have shown to increase the likelihood of AMD in certain individuals [66]. Similarly, Synoweic et al. have shown that G/A genotype transition of the *HMOX2* gene translates to an increased risk of dry AMD, whereas the A/G genotype is protective [67]. The 19th nucleotide position of the *HMOX1* gene is also correlated with reduced risk of AMD. The G/C transversion genotype of the 19th position of the *HMOX1* gene explains the switch from the dry to the wet form of AMD. The 19G>C-*HMOX1* and the −42 + 1444A>G-*HMOX2* mutations are overall considered to aid in the progression of AMD in AMD patients [67]. The reduced protein levels of HO-1 and HO-2 in exudative AMD patients indicate HO-1- and HO-2-targeted degradation. Both HO-1 and HO-2 were found to be more highly concentrated in the lysosome than their usual position in the cytoplasm, indicating the high turnover rate of HO-1 and HO-2 in the oxidatively stressed retinal environment [68]. Young healthy individuals show the opposite: HO-1 and HO-2 display much higher levels in the cytoplasm. Interestingly, glutathione peroxidase, another downstream Nrf2 phase II antioxidant, does not have any apparent activity in the RPE of AMD patients [68].

Furthermore, polymorphisms in the mitochondrial isomer SOD2 have been associated with AMD prevalence. Kimura et al. analyzed the *SOD2* gene from 102 Japanese wet AMD patients and discovered a valine to alanine switch that translates to a ten times higher risk of developing wet AMD. Although glutathione S-transferases (GST) and microsomal epoxide hydrolase exon-4 show possible polymorphisms, the genotype frequency distributions are not as significant [69]. While these results are more indicative of the Japanese AMD population and distribution, and non-Japanese populations are thought to not have these specific *SOD2* polymorphisms associated with AMD [70], the general consensus is that reduced SOD2 levels are associated with AMD progression [57]. New research suggests that epigenetic control of the *SOD2* gene may accelerate AMD progression because mitochondrial dysfunction and H_2_O_2_ accumulation are known to increase oxidative damage and death of RPE cells [71, 72]. Overall, these data in particular supported the generation of multiple Nrf2 pathway knockout animal disease models to further study AMD.

Another study by Huang et al. examined how cigarette smoke, a major risk factor for AMD, induced a UPR response injurious to RPE cells. Although cigarette smoke extract activates the apoptotic pathway, Nrf2 upregulation decreases CHOP, a proapoptotic protein, and confers RPE protection [5].

#### 2.1.4. Nrf2 Pathway-Deficient Animal Models of AMD

Because the Nrf2 pathway is considered to be severely impaired in AMD patients, several disease models involving the knockout of *Nrf2* (*NFE2L2*) and its downstream genes have been proposed and evaluated. *Nrf2* knockout mice display many of the classical signs of AMD pathology, such as soft, large, and yellow drusen-like deposits with distinctly irregular borders [65]. In the peripheral retina, geographic atrophy visible through lesions was found in the RPE. RPE degeneration was also prominent in 12-month *Nrf2* knockout mice, as knockout mice had RPE deficiency, extensive vacuoles, hypopigmentation, and hyperpigmentation. Thickening of the Bruch's membrane was also seen in histological analysis, and staining showed drusen deposition between the RPE and Bruch's membrane. CNV, indicative of the wet form of AMD, was found in nearly 20% of the knockout eyes examined. Considered to be the age pigment, lipofuscin autofluorescence was also found in knockout mice. Zhao et al. further examined the lysosomal degradation pathway to investigate whether RPE cells were capable of phagocytizing the photoreceptor outer segment. Autophagy vacuoles were aberrant and prominent, as enlarged mitochondria were close to autophagy vacuoles [65]. Defects in the lysosomal pathway in *Nrf2* knockout mice show the compromised phagocytic ability of the RPE and also support the idea that cellular homeostasis is breached.

The *SOD1* knockout mouse has also been shown to develop AMD symptoms, with white to yellow drusen-like deposits evident by 10 months of age in 86% of the knockout mice examined [73]. Constant light exposure for 24 h induces drusen formation in young 5-month-old *SOD1* knockout mice, and drusen accumulates with increased exposure time. RPE and Bruch's membrane histology changes are evident, and tight junctions are also lost. Tight junction loss decreases RPE integrity and leads to abnormal RPE morphology, including the loss of its typical hexagonal shape. Additionally, 10% of the *SOD1* knockout mice older than 10 months had observable CNV [73].

Mitochondrial dysfunction has been hypothesized to translate to severe oxidative stress and retinopathy [55, 74]. Because *SOD2* knockout mice do not live beyond 2.5 weeks post birth, the mitochondrial SOD isomer *SOD2*-deficient mice were induced using an AAV-ribozyme-conjugated gene specific for SOD2. The AAV-SOD2 ribozyme particle was injected into the retinas of adult C57BL/6 mice. By four months after injection, *SOD2*-deficient mice had abnormal electroretinograms with 33% and 44% decreases in a- and b-waves, respectively [75]. Hypopigmentation was evident by one month post injection, and outer and inner photoreceptor segments started thinning by 2 months. Extensive vacuoles and RPE atrophy were found to dominate between 2 and 4 months of injection. Electron microscopy further characterized RPE degeneration and thickening of the Bruch's membrane. Also, lipofuscin aggregation was seen by 4.5 months of injection [75].

Although other Nrf2 pathway genes have not been used to define additional AMD animal models, other studies have emphasized that knockout or knockdown of the Nrf2 pathway results in oxidative stress and inflammatory symptoms. For example, *HO-2* knockout mice show abnormal inflammatory processes, impaired corneal healing, ulceration, and choroidal neovascularization [76]. *HO-1* knockout animals have early inflammation and are immunosuppressed [77, 78]. Considering that polymorphisms in several Nrf2 pathway genes are associated with higher AMD risk, more research on antioxidant-deficient models is necessary to uncover the relationship between aging, oxidative stress, and AMD pathogenesis.

#### 2.1.5. Therapeutic Potential of Nrf2 Activation in AMD Treatment

*(1) Small Molecule Nrf2 Activators*. Many studies have used Nrf2 pathway activating drugs to evaluate the cytoprotective role of Nrf2 in retinal tissues and particularly in rescuing oxidation-induced RPE cell damage and death. By conjugating quercetin and phospholipid together to improve bioavailability, Xu et al. were able to increase RPE proliferation by nearly 80%, reduce ROS and MDA levels, and prevent apoptosis by increasing Nrf2 translocation and upregulating Nrf2 downstream proteins such as HO-1 and NQO1 [79]. Using both synthetic and natural flavonoids, Hanneken et al. demonstrated that pretreatment of flavonoids in low concentrations protected against H_2_O_2_-induced oxidative injury by restoring RPE cell viability to about 80–100% [80]. The main protective mechanism involved induction of the Nrf2 pathway and its downstream detoxification proteins.

Certain antioxidant natural products and their analogs can be efficacious in stimulating the Nrf2 pathway. Liu et al. analyzed the potential of a natural product drug, RTA 408, in protecting RPE cells. RTA 408, in nanomolar concentrations, has been previously used to treat multiple cancers and mitochondrial myopathies. The mechanism behind RTA 408's efficacy in increasing cell viability in an H_2_O_2_-stressed environment and restoring redox balance is through the activation of the Nrf2 pathway [81]. With *Nrf2* gene knockdown, RTA 408 cannot exert full RPE protection. Another study by Wang et al. highlights escin, a natural triterpene saponin, as a potential drug to prevent H_2_O_2_-induced RPE damage and death by activation of the Nrf2-AKT signaling pathway. Escin's ability to reduce ROS and increase cell viability was shown by increasing Nrf2 phosphorylation and leading to higher expression of HO-1, NQO1, and SRXN-1 [82]. P13 kinase/AKT pathway can be used to induce Nrf2 activation through phosphorylation [83], yet other studies have evidenced that alternative posttranslational modifications can also regulate Nrf2 stimulation [84]. Liu et al. showed that glutaredoxin 1 (Grx1), a Nrf2-regulated antioxidant enzyme, can deglutathionylate AKT, which in turn can be activated and can reduce oxidative damage in RPE cells [85]. Pinosylvin is another natural product-derived polyphenol from bark that can protect against oxidative damage induced by hydroquinone by upregulating a downstream enzyme in the Nrf2 pathway, HO-1. At 5–10 *μ*M, pinosylvin has antioxidative, anti-inflammatory, and immunomodulatory properties by stimulation of HO-1 [86]. Likewise, salvianolic acid A in RPE cells was shown to phosphorylate and activate Nrf2 and HO-1 via activating AKT/mTORC1 signaling, thus inhibiting H_2_O_2_-induced oxidative stress damage and death [87]. Curcumin, a compound found in turmeric, has also shown multiple antioxidative and anti-inflammatory properties in RPE cells. It protects against both acrolein- and light-induced damages [88, 89]. Interestingly, in other studies, curcumin is controversially thought to be an antiproliferative and possibly cytotoxic agent in RPE cells [90, 91].

*α*-Tocopherol, the most biologically active form of vitamin E, is protective against RPE oxidative injuries. The molecular mechanism behind its RPE protection was examined using acrolein, a lipid peroxidation by-product and constituent of cigarette smoke, as an oxidative stressor. Acrolein causes severe oxidative damage to the RPE cells. However, *α*-tocopherol pretreatment accelerates Nrf2 activation and increases the expression of multiple other antioxidant enzymes, such as GST, HO-1, NQO1, and SOD1. Overall, the Nrf2 pathway proteins exerted cytoprotective effects. Moreover, *α*-tocopherol repairs mitochondrial dysfunction, a contributor to AMD pathogenesis, and restores redox balance in RPE cells [92]. Zinc is also an established vitamin that has antioxidative properties. Zinc reestablishes GSH levels in the RPE to 70% of preinjury level by activating Nrf2, which in turn promotes de novo GSH synthesis [93]. A zinc transporter protein, Zip2, is responsible for transporting zinc into the cell so that the Nrf2 and GSH synthesis pathway could be stimulated [94]. GSH is a crucial small molecule antioxidant that can directly scavenge free radicals. In oxidative stress environments, GSH can conjugate proteins, causing the formation of glutathionylated proteins (PSSG). High PSSG accumulation is often indicative of a deviant redox state and can cause cell damage and death [95, 96]. Hence, chemicals and drugs capable of enhancing GSH levels and restoring redox balance are of key importance to preventing and reducing RPE oxidative injury. In a model of photo-oxidative damage, Gao et al. examined the capability of isothiocyanate sulforaphane in promoting resistance to damaging UV light. Their research highlighted that when Nrf2 pathway is amplified either by *Nrf2* overexpression or by *Keap1* knockdown, GSH and NQO1 levels were boosted in mouse embryonic fibroblasts and conferred protection against UV light damage [97]. Nrf2's capability of promoting cell survival via its antioxidant downstream pathway is due to the scavenging of free radicals and the reinstatement of a good redox balance in the cell.

Other compounds, such as E330 and the NSAID bromfenac, exhibit oxidative stress protection and antiangiogenic properties by stimulating the Nrf2/HO-1 axis. E330 is effective in reducing laser-induced RPE oxidative damage in vivo. This includes preventing CNV and reducing damage to the RPE-Bruch's membrane complex [98]. Bromfenac promotes Nrf2 translocation and upregulates HO-1 expression, which ultimately leads to a decrease in CNV [99].

*(2) Nrf2 Gene Therapy*. Gene therapy is an innovative method to correct or repair faulty or devoid genes. Ildefonso et al. used an AAV vector to introduce a cell-permeable Nrf2-derived peptide (TatNrf2mer) capable of binding Keap1 in the cell. In stably transfected RPE cells, TatNrf2mer restores cell viability by nearly 20% in H_2_O_2_-stressed RPE cells and 50% in paraquat-stressed cells. Moreover, GST, NQO1, and catalase expressions are increased by nearly three-fold in these transfected cells [100]. Intravitreal injection of AAV-TatNrf2mer partially protects photoreceptor function based on ERG responses and optical coherence tomography evaluations in the sodium iodate-induced RPE oxidative mouse model [100]. Moreover, Nrf2 induction showed much lower levels of proinflammatory markers like IL-6 and MCP-1 in a mouse model of uveitis [100]. Inflammation plays a key role in complement deposition and drusen formation [101]. Thus, Nrf2's ability to reduce inflammation and oxidation makes it a potential drug target for treating and preventing AMD.

### 2.2. Cataracts

#### 2.2.1. Cataracts: A Primary Cause of Vision Loss in the World

Cataracts are a form of blurred vision that results from the cloudiness of the lens. Because the light pathway is obstructed, vision loss and blurriness result. It is the most common cause of vision loss in people over the age of 40 around the world, including nearly 20.5 million Americans [102]. Fifty percent of individuals over 80 years old have had or will have cataracts in their lifetime. There are three main types of cataracts: subcapsular, cortical, and nuclear cataracts. Subcapsular cataracts usually involve the breakdown of lens fibers and the accumulation of granular or fibrillary material. Cortical cataracts involve radial or wedge-like opacification, particularly in the cortex area. Nuclear cataracts are the most common type of cataracts and involve the yellowing of the lens nucleus. Along with various mechanisms, each type of cataract has different associated risk factors [102]. Nonetheless, aging and oxidative stress (such as UV irradiation) are the common denominators. However, use of corticosteroids, diabetes, and lifestyle choices such as malnutrition, sunlight exposure, smoking, and alcohol consumption can increase the likelihood of cataract formation [103]. Currently, the only successful treatment for cataracts is surgery, but with a rapidly aging population, there is a growing need to find alternative treatments to prevent and treat cataracts [104].

#### 2.2.2. Maintenance of Transparency in the Lens

In order to keep a clear pathway for light penetration to the retina, the lens must be a flexible, transparent, and biconvex structure [105]. It is composed of the lens capsule, epithelium, and fibers. The transparent lens capsule surrounds the lens and is very elastic with type IV collagen and glycosaminoglycans as its major components. The lens epithelium maintains osmotic volume and forms progenitors for the lens fiber cells. The lens fiber, the bulk of the lens, can stretch anteriorly and posteriorly. To maintain transparency, the lens fiber cells have no organelles or nucleus, and the lens itself has no connective tissue, blood vessels, or nerves [102]. The most common protein found in the lens is crystallins, water-soluble chaperones that aggregate closely together to increase refraction and maintain clarity [106].

#### 2.2.3. Contributions of Oxidative Stress in the Development of Cataracts

Despite chronic exposure to UV light, compared to other tissues, the lens has a competent and efficacious antioxidant system to combat oxidative stress [107, 108]. Particularly, the lens is rich in proteins with sulfhydryl groups that allow for tight packing for refraction and transparency [109]. It is fully equipped with GSH, an antioxidant that can scavenge free radicals. High amounts of enzymes that carry out de novo GSH synthesis allow for continuous production of GSH [110]. When GSH levels start to deplete with age, especially in the nucleus of the lens, protein oxidation can lead to PSSG, protein aggregation, decreased crystallin, protein solubility abnormalities, and an overall yellowing of the lens [111–113]. The outcome is the eventual development of nuclear cataracts.

Oxidative stress plays a major role in the development of cataracts. In nuclear cataracts, it is predicted that the loss of reduced protein sulfhydryl groups is a major mechanism for cataract growth. More than 90% of cysteine residues and 50% of methionine residues are lost or oxidized [103]. Lou et al. first developed the idea of high amounts of protein-mixed disulfides (PSSP) as the main reason for protein aggregation in the lens [114, 115]. Normally, small amounts of PSSP or PSSG serve multiple normal biological functions such as cell signaling. After all, cysteines are often found in the active site of proteins and can control protein functions. However, continuous oxidation of critical cysteines in proteins can render proteins inactive and eventually lead to their aggregation with other proteins. Lou et al. showed that the increasing yellow discoloration corresponds with an increasing concentration of PSSP and PSSG in the lens nucleus [116]. High levels of PSSP and PSSG are a sign of abundant oxidative stress and damage. With increasing oxidative damage, cell death can occur, and the lens will inevitably develop cataract formation.

Moreover, aggregation in cataracts can be mediated by the abnormal activity and loss of *α*-crystallin. Normally, *α*-crystallin works as a chaperone protein that supports lens structure, prevents protein aggregation, and maintains lens protein functionality. Because of increasing protein oxidation and posttranslational modifications like glutathionylation, *α*-crystallin becomes disabled, causing unrestrained protein aggregation and opacity of the lens [109, 111, 112, 117].

Considering that protein aggregation is heavily involved in lens opacity, it is proposed that restoration of redox balance through reversal of PSSP and PSSG formation can be used as both a preventive measure and a treatment for cataracts. The Trx and Grx antioxidant enzyme systems are fully equipped to reverse posttranslational modifications such as PSSP and PSSG and improve the redox balance in the cells [114, 115, 118]. With the reducing power of NADPH, they act as thioltransferases that reduce protein thiols and reactivate proteins. Moreover, de novo GSH synthesis enzymes, glutathione peroxidase, and other vital antioxidant enzymes can scavenge free radicals to prevent excessive protein oxidation [109, 114, 119]. Nrf2 is the major antioxidant transcriptional regulator that controls the transcription of these specific antioxidant systems and enzymes. Decline of Nrf2 in aging and oxidative stress conditions can translate to lower levels of cytoprotective antioxidant enzymes and may cause cataract formation [103, 111, 120].

#### 2.2.4. Involvement of the Nrf2 Pathway in Cataracts

According to von Otter et al., certain *Nrf2* gene mutations can predict the onset of cataracts but do not necessarily confer higher risk to cataract formation. In one study, 489 cataract cases of European ancestry were analyzed for single nucleotide polymorphism and haplotypes at the *Nrf2* and *Keap1* gene locus. Although none of the single nucleotide polymorphisms for *Nrf2* or *Keap1* gene showed predilection for cataracts, one *Nrf2* haplotype GAAAA did determine the advancement of cataract formation by showing a significant correlation of this haplotype with the onset of cataracts four years earlier [121]. However, having haplotype allele GAAGAGGC in the *Nrf2* gene delayed the need for cataract surgery four more years [121]. Because studies have supported that any delay in the onset of cataracts can save billions of dollars and decrease the incidence of cataracts, these findings may be useful in determining how to prevent or treat cataracts.

Despite the lack of correlation between *Nrf2* haplotypes with cataract risk, epigenetic modifications of the *Nrf2* and *Keap1* genes have been predicted to accelerate cataract pathology. In a study by Gao et al., Nrf2 expression was the lowest in the lens epithelium of the 65–80-year-old individuals, whereas Keap1 expression was significantly increased. Levels of mRNA had correspondingly similar results. When methylation of the *Keap1* gene was examined in 45–90-year-old healthy and cataract lens, gradual demethylation was evident with increasing age, from 16% in the 45–65-year-old group to 39% in the 65–80-year-old group. This is comparable to the 42% demethylation found in cataract lens in the 65–80-year-old group [122]. Demethylation often corresponds with an increase in transcriptional activation of *Keap1* gene, which may explain the lower amount of Nrf2 pathway antioxidant proteins with aging and cataract formation. Aging combined with protein oxidation can increase susceptibility to cataracts. Other studies have supported *Keap1* demethylation in diabetic cataractous lenses. Palsamy et al. have shown that in a study of 21 diabetic cataractous lenses, fragment-1 of cataractous lens consisted of 20 CpG dinucleotides, compared to the fragment-2 of clear lens consisted of 43 CpG dinucleotides. Of those CpG islands in cataractous lens, only 12% of the cytosines were methylated, whereas 64% of the cytosine residues were methylated in clear lens [123]. Clear lens had much higher levels of methylation, indicating less transcription of the *Keap1* genes. Moreover, their study showed that in human lens epithelial cell lines, higher *Keap1* methylation corresponded to increased ROS levels and cell death. Another recent study by the same group revealed that methylglyoxal, a major compound produced as a result of high-sugar diets that cause diabetic complications, can inhibit Nrf2 and DNA methyltransferases and upregulate the demethylation enzyme TET1 [124]. Methylglyoxal functions in altering arginine and lysine residues of lens proteins by causing protein aggregation and leading to the formation of advanced glycation end products [125]. This leads to an increased *Keap1* demethylation, increased proteasomal degradation of Nrf2, an elevated UPR, and consequential endoplasmic reticulum (ER) stress [126]. This group has also attributed valproic acid, a common antiseizure drug, in stimulating the demethylation of *Keap1* and increasing the incidence of cataracts in epilepsy patients [126].

Research is currently limited in Nrf2 pathway genes and their involvement in cataract development. Chandra et al. showed that in 131 cataract cases of North Indian people, there was an association between the *glutathione S-transferase mu 1* (*GSTM1*) null genotype with cataract formation. The frequency of the *glutathione S-transferase theta 1* (*GSTT1*) gene was also found to be 20% less in cataractous lenses [127].

#### 2.2.5. Nrf2 Pathway-Deficient Animal Models of Cataracts

Although there has not been an established *Nrf2* knockout model for cataracts, there have been models that use pro-oxidants to suppress Nrf2 activation. Palsamy et al. injected sodium selenite into the lenses of rats and observed nuclear cataract formation by the 5th day. Demethylation of the *Keap1* gene was induced by selenite and led to decreased transcription of the Nrf2 antioxidant pathway. Due to diminished calcium homeostasis, low ATP levels, reduced GSH levels from impaired Nrf2 function, ROS overproduction following UPR activation, and ER stress all generated cell damage and death [126].

Phase II antioxidant gene knockout models have also been generated to accelerate cataract formation. Regulated by Nrf2 activation, the Grx family is composed of two specific subsets: the cytoplasmic Grx1 and the primarily mitochondrial Grx2. The Grx family is heavily involved in reversing PSSG and reducing apoptosis in an oxidatively stressed environment. It is concentrated throughout ocular tissue and is thought to reduce light-induced oxidative damage particularly in the lens [128, 129]. Considering the part that PSSG plays in aggravating oxidative stress, protein aggregation, and opacity in the lens, Lou et al. developed two cataract models using both *Grx1* and *Grx2* knockout mouse models [107, 130].

Lens epithelial cells cultured from *Grx1^−/−^* mice have rounder shape and increased volume compared to the normal elongated form in *Grx1^+/+^* mice. Cell migration is severely impaired, which correlates with an increased doubling time for cell proliferation in *Grx1^−/−^* mice. Primary lens epithelial cells isolated from *Grx1^−/−^* mice were much more sensitive to oxidative stress as compared with normal lens epithelial cells [130].

Wu et al. characterized the *Grx2^−/−^* mouse as a potential cataract model. Because mitochondria are heavily involved in ROS production, mitochondria dysfunction is common in oxidative stress-induced diseases such as cataracts [107]. The primary mitochondrial location of Grx2 makes it a protein of interest especially due to recent publications defining Grx2's antiapoptotic abilities, direct peroxidase activity, and protection over key electron transport chain proteins including complexes I and IV [131, 132]. These studies showed that *Grx2* gene deletion accelerates cataract pathogenesis, including augmenting PSSG formation and diminishing mitochondrial function and ATP production. By 11 months of age, 80% of *Grx2^−/−^* mice had developed severe cataracts, whereas only 20% of wild-type mice did [133]. By 16 months, GSH level drops to about 33%, and protein sulfhydryl level drops to about 30% in knockout mice. ATP production is completely devoid in 16-month-old knockout mice, which is accompanied by higher glutathionylation of complex I and complex IV [132–134].

#### 2.2.6. Therapeutic Strategies Based on the Nrf2 Pathway for Cataracts

For diabetic patients, chronic inflammation and oxidative stress can progress to diabetic cataracts [103]. A study by Liu et al. examined *Rosa laevigata* Michx. extract as a potential natural product drug to treat high glucose-stressed lens epithelial cells. HO-1 induction via Nrf2/AKT signaling was responsible for its cytoprotective effects by lowering ROS and increasing mitochondrial membrane potential [135]. DL-3-n-butylphthalide (NBP), an anti-inflammatory and antiapoptotic neuroprotective drug, was examined to see its effects in treating cataracts in streptozotocin-injected rats. ROS and lipid peroxidation products like MDA were found to be much lower in drug-treated diabetic rats. Cataract onset without treatment was seen by 3 weeks and mature cataracts by 9 weeks in diabetic rats. With NBP, cataract onset was significantly delayed, only starting by 6 weeks [136]. By 9 weeks, mature cataracts were not present in diabetic rats with NBP treatment. Nrf2, Trx, and catalase were all upregulated with NBP treatment, indicating that Nrf2 activation is partially responsible for the prevention of cataract pathology in diabetic rats [136].

Vitamin deficiency has also been attributed to cataract formation [102]. Homocysteine is a common pro-oxidant that can cause many inflammatory disorders including heart attack and stroke. Yang et al. analyzed the neuroprotectant and dietary supplement acetyl-l-carnitine as a possible candidate to treat homocysteine-induced lens epithelial cell damage. Acetyl-l-carnitine demonstrated antiapoptotic abilities and decreased ER stress by stimulating Nrf2 and several of its phase II antioxidants including GSH, catalase, SOD, and glutathione peroxidase [137]. The isothiocyanate sulforaphane is another dietary supplement found in cruciferous vegetables like cabbage and horseradish. In addition to an anticarcinogenic function, sulforaphane is also known to induce detoxification and antioxidant enzymes. Using H_2_O_2_ as a stressor, Liu et al. showed that sulforaphane pretreatment in human lens epithelial cell line FHL124 reduced apoptosis and DNA damage by upregulating Nrf2 translocation into the nucleus [138]. Varma et al. further discovered that sulforaphane is capable of inciting Trx activity 18 times as much compared to nontreated mice lens cells [139]. Trx is vital for the reduction of PSSPs and improving the redox balance of the lens.

Because *Keap1* methylation is accredited to inhibiting Nrf2 activation and is found in higher amounts of cataract patients, drugs increasing methylation or decreasing demethylation are being evaluated for their aptitude in restricting cataract pathology [123, 124, 126]. Gene therapy is also being considered and may represent the future in preventing and treating oxidative stress-induced lens abnormalities.

### 2.3. Diabetic Retinopathy

#### 2.3.1. Epidemiology of Diabetic Retinopathy

Diabetic retinopathy (DR) is the most common retinal vascular disease and the leading cause of new cases of blindness in adults [140]. The number of people afflicted with this complication of diabetes is expected to reach 15 million by the year 2050 [140]. It is estimated that DR may soon become the leading cause of visual impairment in the world. The retina uses more oxygen than any other tissue in the body on a per unit weight basis, thus making it very susceptible to oxidative stress. Diabetes alters the balance between the oxidant-antioxidant system by increasing ROS as well as compromising the antioxidant defense system [141]. An increase in ROS is critical in the development of diabetic complications, specifically DR [142]. The highly oxygenated environment of the retina combined with the impaired redox homeostasis seen in diabetes is a crucial prerequisite for the development of DR [142].

#### 2.3.2. Contribution of Oxidative Stress in Diabetic Retinopathy

The possible sources of oxidative stress in diabetes include auto-oxidation of glucose, enhanced aldose reductase activity, increased advanced glycation end products, and altered protein kinase C activity [143]. Together, these various pathways culminate in the production of ROS that upsets the body's redox homeostasis [142]. Additionally, hyperglycemia, hyperlipidemia, and inflammation are the three main metabolic abnormalities in diabetes, all of which are able to stimulate generation of ROS and inflict oxidative stress [140]. Studies have shown that oxidative stress develops in the retina of diabetic animals and galactose-fed animals [144]. This is illustrated by the evidence that nondiabetic animals, in which blood hexose concentration is increased with a galactose-rich diet, develop retinal capillary lesions that are identical to those that develop in diabetic humans or animals [144]. These findings indicate that oxidative stress is at least associated with the development of DR.

#### 2.3.3. Involvement of Nrf2 Pathway in Diabetic Retinopathy

The Nrf2-Keap1 system plays an integral role in the multifaceted pathophysiology of DR. On a cellular level, Nrf2 has shown to be localized in multiple cell types within the retina [141]. Immunohistochemistry experiments have disclosed prominent Nrf2 staining in Muller cells [141]. The Muller cell is an important driver of the proinflammatory processes involved in the progression of DR, including the generation of superoxide radicals [141].

In the retina, the role of Nrf2 is to act as a cytoprotective mechanism in response to oxidative stress [141]. Protection from this injury is crucial to avoid ocular damage by ROS generated in the pathogenesis of diabetes. However, the elevated states of glucose in diabetes decrease the protection offered by Nrf2 [141]. This is supported by a study that found a significant increase in retinal superoxide in diabetic mice deficient in Nrf2 compared with wild-type mice after 5 weeks of diabetes [141]. In addition, the level of the intracellular antioxidant GSH is reduced in diabetes [145]. The enzymes responsible for the GSH redox cycle (glutathione peroxidase and glutathione reductase) are also compromised [145]. GSH biosynthesis is an integral part of the protection system against oxidative stress, and glutamate cysteine ligase is the rate-limiting enzyme in this biosynthesis reaction [145]. Nrf2 is considered a key transcription factor for the regulation of the catalytic subunit of glutamate cysteine ligase (GCLC) [145]. The site of histopathology associated with diabetic retinopathy are the retinal endothelial cells (RECs), and quantification of the Nrf2-GCLC pathway in isolated RECs revealed that the nuclear expression of Nrf2- and DNA-binding activity were decreased by 50% to 60% in RECs exposed to high glucose levels [145]. Binding of Nrf2 with GCLC was also decreased by 90% in diabetic rats, resulting in a significant decrease in GCLC expression [145]. This demonstrates that high glucose levels impair Nrf2 activity and result in a blunted expression of the antioxidant gene GCLC. Studies of human donor eyes with DR also showed decreased GCLC levels compared with age-matched nondiabetic controls [145]. Further evidence shows that disturbances in Nrf2-GCLC signaling are a major event in the development of DR as indicated by the observation that diabetic Nrf2 knockout mice exhibited a significantly lower retinal GSH level compared with wild-type diabetic mice [141, 145]. Although the precise mechanism by which diabetes affects Nrf2-mediated regulation of GSH biosynthesis remains to be elucidated, the inverse relationship between glucose levels and GCLC activity is clearly evident by these observations. This is an important conclusion because it explains the pathway that begins with hyperglycemia and leads to a weakened antioxidant defense system, specifically in the retina. A suboptimal antioxidant defense system due to low retinal GSH sets the stage for the development of DR.

Immunofluorescence studies reveal that under high glucose levels, cytosolic expression of Nrf2 was increased along with increased localization of both Nrf2 and Keap1 in the cytosol [141, 145]. As discussed earlier, Keap1 normally sequesters Nrf2 in the cytosol, which blocks the transcription of antioxidant genes. Diabetic rats have been shown to express increased mRNA and protein levels of Keap1 compared to those from normal control rats [145]. To confirm these in vivo findings, RECs were transfected with Keap1-siRNA, which prevented a glucose-induced decrease in Nrf2 accumulation in the nucleus [145]. These observations illustrate that the hyperglycemic environment results in increased expression of Keap1, thereby leading to increased cytosolic sequestration of Nrf2. This study also suggests that Keap1 knockdown can play a role in allowing Nrf2 to translocate to the nucleus and initiate transcription of antioxidant genes.

The findings reported here concerning the Nrf2-Keap1 system in DR suggest that increased oxidative stress created by the diabetic environment prevents Nrf2 from reaching the nucleus to enhance the transcription apparatus. A redox reaction of the Cys-151 cysteine residue of Keap1 is a key modification required for Nrf2 to dissociate from Keap1 and enter the nucleus [146]. It is possible that the oxidative stress in diabetes alters the redox-sending capacity of Keap1, thereby preventing the dissociation of Nrf2 from Nrf2-Keap1 complex [146]. Another explanation of the altered binding between Nrf2 and Keap1 in diabetes includes diabetes-induced posttranslational or epigenetic modifications of retinal proteins, including Keap1 [147–149]. In fact, nitration, ribosylation, and other posttranslational modifications of retinal proteins have been shown to occur in diabetes [123]. In addition, epigenetic modification of lens Keap1 has also been shown in diabetes [123].

The protective role of Nrf2 is further exemplified by evidence that Nrf2 knockout mice show an increase in inflammatory mediators regardless of blood glucose level [143]. Inflammation is independently associated with an increase in vascular damage in DR and also BRB breakdown [150]. Accumulating evidence shows that inflammation is the key mediator of the endothelial cell injury and BRB dysfunction [150]. TNF*α* (tumor necrosis factor-*α*) is an inflammatory cytokine that plays an important role in DR, including BRB dysfunction [150]. Xu et al. have shown a significantly greater amount of TNF*α* protein in diabetic mice deficient in Nrf2 compared to wild-type diabetic mice [141]. Since an increase in TNF*α* is associated with breakdown of the BRB, it follows that Nrf2 knockout mice exhibited a significant increase in retinal vascular leakage after 8 weeks of diabetes compared with wild-type diabetic mice [141]. Retinal edema and loss of vision are the direct results of retinal vascular leakage; this pathology is a major end point of DR [141]. These findings indicate the integral role Nrf2 plays in mitigating proinflammatory cytokines, regardless of blood glucose levels.

Visual impairment is a well-regarded end point of diabetes due to the adverse effects on neuronal function caused by hyperglycemia [151]. Since Nrf2 has neuroprotective effects in the retina, Xu et al. measured the effects of Nrf2 on diabetes-induced visual dysfunction in mice using the parameters of spatial frequency and contrast sensitivity [141]. They found that after 8 weeks of diabetes, Nrf2 knockout mice had significant visual deficits in both spatial frequency and contrast sensitivity as compared to nondiabetic Nrf2 knockout controls and diabetic wild-type mice [141]. This finding suggests that Nrf2 deficiency exacerbates diabetes-induced visual impairment in mice.

Diabetes skews the balance between oxidative stress and the antioxidant system. A hyperglycemic environment creates a state of increased oxidative stress in tissues of both humans and animals, and increased oxidative stress might play a role in the development of diabetic complications. Increase in ROS is one of the major retinal metabolic abnormalities associated with the development of DR. The studies presented earlier demonstrate a common theme: the Nrf2-Keap1 signaling pathway plays an important role in the pathogenesis of diabetic retinopathy. A hyperglycemic environment and subsequent oxidative stress adversely affect the binding between Nrf2 and Keap1 and Nrf2 and GCLC, and these also contribute to visual dysfunction. Visual dysfunction is not only caused by a hyperglycemic state, but it is also exacerbated by a loss of Nrf2 due to hyperglycemia. This discussion suggests that Nrf2 is protective against both oxidative stress and inflammation, and hyperglycemia leads to loss of cytoprotection offered by Nrf2.

#### 2.3.4. Therapeutic Strategies Based on the Nrf2 Pathway for DR

At this time, we did not find any published study showing the use of Nrf2-related compounds as a therapeutic approach to treat DR. A recent study by Nakagami et al., however, succeeded in showing that a novel activator of Nrf2 known as RS9 delayed retinal degeneration by inhibiting inflammatory responses and increasing intrinsic antioxidant enzymes [152]. Increase in inflammation and oxidative stress are the cornerstones of the pathophysiology of DR. Thus, this study supports Nrf2 activators' role as possible therapeutic agents for DR.

### 2.4. Glaucoma

#### 2.4.1. Glaucoma Pathophysiology

Glaucoma is a group of diseases characterized by optic neuropathy and retinopathy. Although the pathogenesis of glaucoma remains uncertain, elevated intraocular pressure (IOP) due to impaired outflow of the aqueous humor is considered the most important risk factor for the disease. The aqueous humor is produced by the ciliary body and drained through the trabecular meshwork (TM). The TM failure reduces outflow of the aqueous humor and increases IOP [153]. Visual loss in glaucoma, characterized by a specific pattern of visual field defects, results from retinal ganglion cell (RGC) apoptosis which appears to be initiated at the optic nerve head where RGC axons pass through.

#### 2.4.2. Involvement of Oxidative Stress in Glaucoma

Increasing clinical evidence suggests that oxidative stress contributes to the pathogenesis of glaucomatous neurodegeneration. Oxidative DNA damage, demonstrated by increased level of 8-hydroxy-2′-deoxyguanosine, has been found in TM tissues from patients with glaucoma [154]. Further study showed the correlation between TM DNA oxidative damage and mean IOP as well as visual field defects in glaucoma patients [155]. Additional evidence supporting the role of oxidative damage in TM degeneration includes significantly decreased total reactive antioxidant potential and increased activities of antioxidant enzymes such as SOD and glutathione peroxidase in the aqueous humor from glaucoma patients [156]. Oxidative stress is demonstrated not only in TM but also in the retina. Upregulation of hypoxic stress-induced proteins, such as hypoxia-inducible factor-1*α* and heat shock proteins, have been shown in the glaucomatous human retina and optic nerve head [157, 158]. These findings highlight the importance of oxidative stress, the cause or consequence of the glaucomatous neurodegeneration, in the pathogenic cascade of the disease.

Genetic factors contribute to the development of glaucoma. Mutations of genes associated with oxidative stress have been correlated with glaucoma. For example, mutations of *CYP1B1*, a member of cytochromes P450 superfamily, have been identified in primary congenital glaucoma [159–161]. The *GSTM1* null genotype appears more frequent in glaucoma patients than in controls in the Mediterranean region [154, 162]. The GSTM1 enzyme is one of the major polymorphisms of GST. These data further support the possible role of oxidative stress in the pathogenesis of glaucoma.

#### 2.4.3. Involvement of Nrf2 Pathway in Glaucoma

The Nrf2 antioxidant response element pathway-mediated neuroprotection has been shown in animal models mimicking certain aspects of glaucoma pathology. Depletion of *Nrf2* aggravates RGC death induced by optic nerve injury [163]. Similar results have been reported in mice following retinal ischemia/reperfusion insult. Compared to wild-type controls, *Nrf2* knockout mice exhibit an exacerbation of oxidative stress, and capillary and neuronal degeneration [164].

#### 2.4.4. Therapeutic Strategies Based on the Nrf2 Pathway for Glaucoma

Consistent with the findings that Nrf2 is potentially involved in glaucoma, activation of endogenous Nrf2 pathway in the retinas using triterpenoid 2-cyano-3,12-dioxooleana-1,9-dien-28-imidazolide, a Nrf2 activator, promotes neuronal survival in both the retinal ischemia/reperfusion and the optic nerve injury models [163, 165]. The Nrf2 pathway appears to be a promising target for glaucoma neuroprotection.

## 3. Therapeutic Outlook

### 3.1. Strategies to Augment Nrf2 Activity

Increase in Nrf2 activity is expected to be protective against oxidative and inflammatory injuries. To achieve that, various approaches have been tried to modulate components of the Nrf2-Keap1 pathway. As mentioned above, compounds that increase Nrf2 activity were evaluated for their therapeutic effects in some of these ocular diseases. For example, quercetin analog, RTA 408, escin, pinosylvin, salvianolic acid A, and curcumin were tested in AMD models; *Rosa laevigata* Michx. extract, NBP, acetyl-l-carnitine, and sulforaphane were assessed in cataract models. Similarly, many natural and synthetic compounds have been assessed in other biological systems for their potential to activate Nrf2 [66, 166, 167]. Discoveries in these biological systems may guide the development of useful drugs for ophthalmology.

In addition to chemical Nrf2 activators, overexpression of Nrf2 is another efficacious means to protect against oxidative stress. Selective overexpression of Nrf2 in the appropriate cells may prevent or reverse ROS-mediated toxicity. This approach has been investigated in various systems [66, 167]; it is likely useful for ocular uses. Similarly, since Keap1 is a negative regulator of Nrf2, interfering with Keap1 activity is a reasonable approach to increase Nrf2 activity, especially in the hyperglycemic environment of the cell. This could be achieved by using siRNA or shRNA directed against Keap1. Keap1 knockdown confers persistent Nrf2 activation and protection against oxidative damage in astrocytes [168], cortical neurons [169], and hepatocytes [170]. It is interesting to note that a number of natural products such as synthetic triterpenoids [171], salvianolic acids [87], and sulforaphane [166] have been identified as potent Nrf2 activators. Instead of directly enhancing the expression level of Nrf2, these compounds inactivate Keap1 by covalently modifying reactive cysteine residues in Keap1, thereby activating the Nrf2 signaling pathway and its downstream target genes. Consequently, activated Nrf2 bypasses Keap1 and then Nrf2 translocates into the nucleus. These results suggest that Keap1 knockdown may be meaningful therapeutic approaches in the future.

In addition to being potential therapeutic means, we speculate that Nrf2-targeted approaches can be useful as disease prevention. By directly stimulating phase II enzyme expression, thereby making cells more resistant to oxidative stress-induced cell injury, preventing ROS overproduction, and inhibiting protein oxidation, Nrf2 activators may be a novel approach for the prevention of a wide variety of oxidative stress-related human diseases and should be used in early or presymptomatic stages of diseases when restoring homeostasis is critical.

### 3.2. Word of Caution: The Potential Dark Side of Nrf2

Nrf2 has many antioxidative and anti-inflammatory functions, which are typically considered to be beneficial for cell survival and proliferation. However, evidence also indicates that small amounts of ROS are essential to induce cell signaling events including insulin and growth factor signaling [96, 172]. Due to the cytoprotective nature of the Nrf2 pathway, it is not surprising that Nrf2 deficiency may make cells more vulnerable to carcinogens. However, a recent study by DeNicola et al. showed that the expression of classic oncogenes, such as *Kras*, *Braf*, and *Myc*, reduced ROS levels by activating Nrf2 pathway and thereby promoting tumorigenesis [173]. In the eye, Pan et al. showed that a small amount of H_2_O_2_ enhances rabbit corneal epithelial cell viability, migration, adhesion, and attachment to the extracellular matrix, as well as improves cornea wound closure in an ex vivo porcine model and an in vivo mouse model [174]. Furthermore, Zucker et al. demonstrated that in NIH3T3 cells, an excessive oxidative stress environment can lead to Nrf2-dependent activation of Kruppel-like factor 9 (Klf9), a ubiquitously expressed protein that regulates cell differentiation and promotes oxidative stress-induced cell death [175]. When ROS exceeds a critical threshold, Nrf2 binds to AREs of the *Klf9* gene and upregulates Klf9 expression. Klf9 in turn suppresses expression of Trx reductase 2, amplifies ROS production cascade, and eventually causes cell death [175]. Therefore, although ROS is generally considered to be detrimental to tissues and beneficial to Nrf2, their roles can be complex. Consequently, it is important to define the boundary between beneficial and potentially damaging effects of Nrf2 activation and determine the appropriate redox balance to maintain healthy cellular homeostasis.

## 4. Concluding Remarks

Inflammation and oxidative stress are important parameters in the pathophysiology of the major ocular diseases. Nrf2 has been shown to have both antioxidant as well as anti-inflammatory properties. These protective effects can be augmented by pharmacologic or molecular modulations. Augmentation of the antioxidant properties and anti-inflammatory properties can provide novel and useful therapeutic targets for these devastatingly blinding diseases.

## Abbreviations

AMD: Age-related macular degeneration
ARE: Antioxidant response element
BRB: Blood-retina barrier
CNV: Choroidal neovascularization
DR: Cataract, diabetic retinopathy
ER: Endoplasmic reticulum
GCLC: Catalytic subunit of glutamate cysteine ligase
Grx: Glutaredoxin
GSH: Glutathione
GST: Glutathione S-transferase
HO: Heme oxygenase
IOP: Intraocular pressure
Keap1: Kelch-like ECH-associated protein 1
Klf9: Kruppel-like factor 9
MDA: Malondialdehyde
NBP: DL-3-n-butylphthalide
NQO1: NAD(P)H:quinone oxidoreductase 1
Nrf2: Nuclear factor erythroid-2-related factor 2
PSSG: Glutathionylated protein
PSSP: Protein-mixed disulfide
REC: Retinal endothelial cell
RGC: Retinal ganglion cell
ROS: Reactive oxygen species
RPE: Retinal pigment epithelium
SOD: Superoxide dismutase
TM: Trabecular meshwork
TNF*α*: Tumor necrosis factor-*α*
Trx: Thioredoxin
UPR: Unfolded protein response
VEGF: Vascular endothelial growth factor.
